# Dynamic model with factors of polycystic ovarian syndrome in infertile women

**DOI:** 10.18502/ijrm.v17i4.4548

**Published:** 2019-06-13

**Authors:** Somayeh Ghiasi Hafezi, Mohammadreza Ahmadi Zand, Mohammadreza Molaei, Maryam Eftekhar

**Affiliations:** ^1^ Department of Mathematics, Yazd University, Yazd, Iran.; ^2^ Mahani Mathematical Research Center and Department of Pure Mathematics, Faculty of Mathematics and Computer, Shahid Bahonar University of Kerman, Kerman, Iran.; ^3^ Research and Clinical Center for Infertility, Yazd Reproductive Sciences Institute, Shahid Sadoughi University of Medical Sciences,Yazd, Iran.

**Keywords:** *Infertility*, * Polycystic ovary syndrome*, * In vitro fertilization*, *
Asymptotically stable*, * Differential equation*, * Free equilibrium point*

## Abstract

**Background:**

Previous studies present various methods for prediction disease based on statistics or neural networks.These models use statistics and results from past procedures to provide prediction through probability analysis.

**Objective:**

In this article, the authors present a dynamic model aiming at predicting the treatment result of infertile women with the factor of polycystic ovary syndrome.

**Materials and Methods:**

For this purpose, the authors have divided the study population into five groups: women prone to infertility, PCOS women, infertile women undergoing the treatment with Clomiphene Citrate and Gonadotropin, infertile women under IVF treatment, and improved infertile women. Therefore, the authors modeled the disease in infertile women mathematically and indicated that the free equilibrium point was asymptotically stable. Also the possibility of other equilibrium point of the system has been studied.

**Results:**

The authors showed that this equilibrium point was marginally stable. Using Stoke's Theorem, the authors proved that the recurrence of the disease cycle with the factor of polycystic ovary syndrome was not intermittent in infertile women. They solved this model numerically using Rung-Kutta method and sketched the figures of the resulted solutions.

**Conclusion:**

It shows that with increasing age, the ovarian reserve is decreased and the treatment Clomiphene Citrate and Gonadotropin are not responsive, so IVF treatment is recommended in this group of patients considering the graphs of the model.

## 1. Introduction

Infertility is referred to the inability of a woman to become pregnant after at least one year of regular sexual intercourse without using birth control. Infertility is seen in 10–15% of couples (1). The main causes of infertility include: ovulatory dysfunction, tuboperitoneal pathology, male factors, and uterine pathology (2). One of the most common causes of ovulatory dysfunction is the polycystic ovary syndrome. In 2003 Rotterdam, the presence of two out of three criteria for diagnosis of PCOS is essential. (i) Clinical and/or biochemical signs of hyperandrogenism, (ii) Oligomenorrhea or anovulation, and (iii) polycystic ovaries (with the exclusion of related disorders) (3). In most cases, women with PCOS have reduced fertility due to low ovulation; therefore, the ovarian stimulation methods are used for a group of these patients due to their reduced ovarian response. Clomiphene Citrate is the major component of the ovarian stimulation treatment, causing 80–85% of the ovulation and leading to pregnancy in 40% of women (2) Gynecologists suggest that after a maximum of six months being in ovulation cycles with the use of Clomiphene Citrate, it is better for the patient to be treated with gonadotrophin. If the treatment cycles with Clomiphene Citrate and Gonadotropin are determined and its therapeutic follow-up is controlled, and yet the pregnancy does not occur during the 9-12 treatment cycles, the ovulation stimulation method should not be relied upon. Therefore, using the Assisted Reproductive Techniques (ART), one of which is IVF, is recommended. Here, we present a model for women infertility with polycystic ovary syndrome, based on the individuals who went to the Yazd Infertility Research and Treatment Clinic. To this end, we show the total number of the infertile couples who at the time of *t* went to the clinic over the course of a year, with *N*(*t*). We have divided them into five groups, including: the newcomers who went to the clinic and their disease has not yet been confirmed, patients (those whose disease has been diagnosed to be and should be treated by this clinic), those treated with Clomiphene Citrate and Gonadotropin, those treated with ART, and improved patients (those whose test was positive). We call this our model.

In the second section, we model infertility in women with *PCOS* using dynamic system methods and find the number of the secondary sufferers during the course of the disease that had returned to the disease, and we show it with ${𝑅}_{0}$.

In the third section, we obtain the equilibrium point of the model ${\mathrm{𝑆𝐼𝑇}}_{1}{𝑇}_{2}𝑅$, using geometric methods and examine its stability, asymptotically.

In the fourth section, we examine the stability of the disease asymptotically in the ${𝑄}_{*}$ equilibrium point.

In the fifth section, using the Stoke's Theorem, we examine the non-intermittence of the therapeutic cycle.

In the sixth section, the model is solved numerically by using Rung-Kutta method, and the obtained data are summarized in the several graphs, which are analyzed in detail.

## 2. Materials and Methods

According to the previous section, we show the total number of the infertile women at time 𝑡 with 𝑁(𝑡). Therefore, $𝑁\left(𝑡\right)=𝑆\left(𝑡\right)+𝐼\left(𝑡\right)+{𝑇}_{1}\left(𝑡\right)+{𝑇}_{2}\left(𝑡\right)+𝑅\left(𝑡\right)$.

Suppose α and γ refer to the birth rate of the disease (the rate of the patient's arrival at the clinic to diagnose and treat their disease) and the death rate (leaving the clinic without any result or not being treated), respectively. By `not being treated', we mean those who were checked at the clinic, and the type of their treatment was diagnosed, but they discontinued their treatment. The rate of abortion and returning to the cycle of treatment is β. The treatment speed of those who have become pregnant through medication (clomiphene Citrate and gonadotropin) is η, and ζ is the treatment speed of those who underwent 𝐼𝑉𝐹 treatment, and their 𝐵𝐻𝐶𝐺 tests were positive.

**Figure 1:**
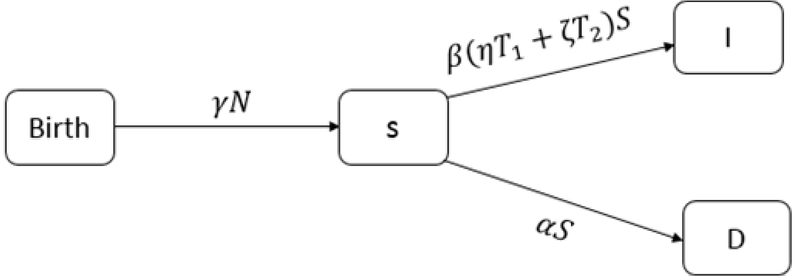
Representation of the susceptible couples.

We show the mean of the infertile people who had abortions or were with 𝑂𝐻𝑆𝑆 disease and returned to the treatment cycle at time 𝑡 by β𝑁(𝑡). The probability of a disease returning in the susceptible woman is equal to $\frac{𝑆(𝑡)}{𝑁(𝑡)}$ (4). Therefore, the number of new women patient per time unit is equal to $\beta 𝑁\frac{𝑆(𝑡)}{𝑁(𝑡)}$, and the number of infertile women who have the possibility of returning to the disease at time 𝑡 is equal to $\beta (\eta {𝑇}_{1}+\zeta {𝑇}_{2})𝑆$. 𝐷 indicates the number of people who left the clinic without obtaining any result from the treatment or without being treated. The number of people prone to the disease entering group 𝐷 is α𝑆 (Figure 1).

Therefore, ${𝑆}^{{}^{'}}=\gamma 𝑁-\alpha 𝑆-\beta \left(\eta {𝑇}_{1}+\zeta {𝑇}_{2}\right)𝑆$.

**Figure 2:**
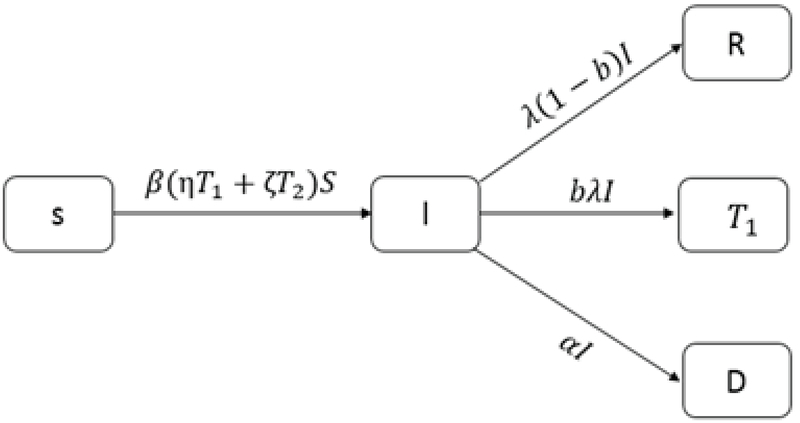
Patient couples are divided to three groups.

We show the treatment rate in the patient group with λ and the treatment rate of λ𝐼 who were under medical treatment with 𝑏. Therefore, the number of patients who used 𝐼𝑉𝐹 treatment is equal to (1-𝑏)λ𝐼. The number of people who left the clinic without any treatment is α𝐼 (Figure 2).

Hence ${𝐼}^{{}^{'}}=\beta \left(\eta {𝑇}_{1}+\zeta {𝑇}_{2}\right)𝑆-\left(\alpha +\lambda \right)𝐼$.

**Figure 3:**
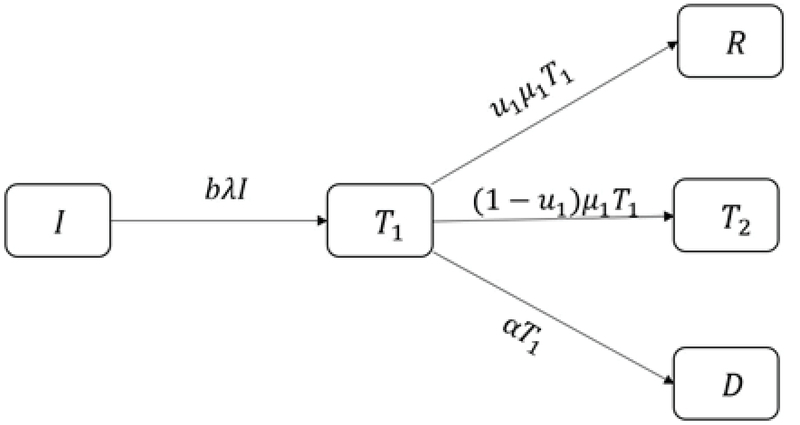
Infertile women under drug treatment.

We show the recovery rate in group ${𝑇}_{1}$ with $\mu_{1}$. We divide $\mu_{1}{𝑇}_{1}$ into two groups: the first group is the number of people who have recovered, and we show it with ${𝑢}_{1}\mu_{1}{𝑇}_{1}$, in which ${𝑢}_{1}$ is the recovery rate of $\mu_{1}{𝑇}_{1}$ at time 𝑡. Therefore, $\left(1-{𝑢}_{1}\right)\mu_{1}{𝑇}_{1}$ of these people enters ${𝑇}_{2}$ group. The number of people under medical treatment who left the clinic without any treatment is $\alpha {𝑇}_{1}$ (Figure 3).

Thus ${𝑇}_{1}^{{}^{'}}=\lambda \mathrm{𝑏𝐼}-\left(\alpha +\mu_{1}\right){𝑇}_{1}$.

**Figure 4:**
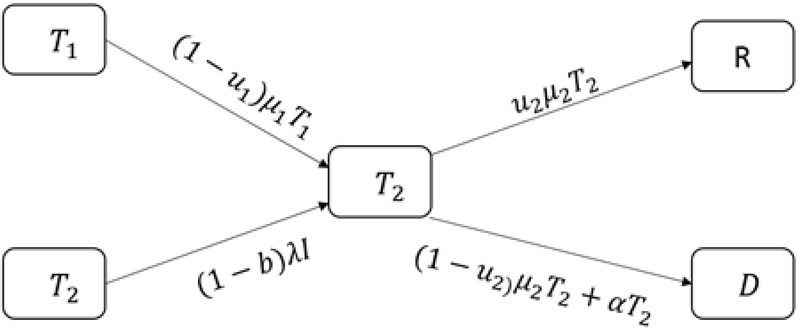
Infertile women under 𝐼𝑉𝐹 treatment.

We show the recovery rate in group ${𝑇}_{2}$ with $\mu_{2}$. The recovery rate of $\mu_{2}{𝑇}_{2}$ at time 𝑡 is ${𝑢}_{2}$, therefore, the number of people treated with 𝐼𝑉𝐹 is equal to ${𝑢}_{2}\mu_{2}{𝑇}_{2}$ and the number of people under treatment entering group 𝐷 is equal to $\left(1-{𝑢}_{2}\right)\mu_{2}{𝑇}_{2}$. The number of 𝐼𝑉𝐹 patients who left the clinic without any result is $\alpha {𝑇}_{2}$ (Figure 4).

According to Figure 4, we have: ${𝑇}_{2}^{{}^{'}}=\lambda \left(1-𝑏\right)𝐼+\left(1-{𝑢}_{1}\right)\mu_{1}{𝑇}_{1}-\left(\alpha +\mu_{2}\right){𝑇}_{2}$.

**Figure 5:**
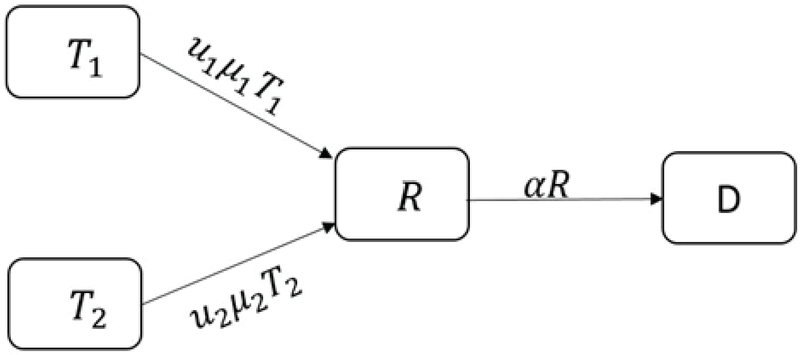
Cured infertile couples.

The number of susceptible people who left the group due to death is α𝑅(Figure 5).

Hence ${𝑅}^{{}^{'}}={𝑢}_{1}\mu_{1}{𝑇}_{1}+{𝑢}_{2}\mu_{2}{𝑇}_{2}-\alpha 𝑅.$

**Figure 6:**
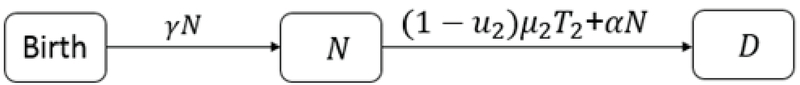
All of the infertile couples.

The number of infertile women who used cure methods is denoted by γ𝑁, and those entered susceptible at 𝑡 time by $\left(1-{𝑢}_{2}\right)\mu_{2}{𝑇}_{2}$ and α𝑁 denotes the number of infertile women who leave the population at 𝑡 time (Figure 6).

According to the aforementioned cases, the dynamic model of infertile patients with 𝑃𝐶𝑂𝑆 can be modeled as follows: 

$$\begin{array}{c} white{𝑆}^{{}^{'}}=\gamma 𝑁-\alpha 𝑆-\beta 𝑆\left(\eta {𝑇}_{1}+\zeta {𝑇}_{2}\right)\\ white{𝐼}^{{}^{'}}=-\lambda 𝐼-\alpha 𝐼+\beta 𝑆\left(\eta {𝑇}_{1}+\zeta {𝑇}_{2}\right)\\ white{{𝑇}_{1}}^{{}^{'}}=-\mu_{1}{𝑇}_{1}-\alpha {𝑇}_{1}+\lambda \mathrm{𝐼𝑏}\\ white{{𝑇}_{2}}^{{}^{'}}=-\mu_{2}{𝑇}_{2}-\alpha {𝑇}_{2}+\left(1-{𝑢}_{1}\right)\mu_{1}{𝑇}_{1}+\left(1-𝑏\right)\lambda 𝐼\\ white{𝑅}^{{}^{'}}=-\alpha 𝑅+{𝑢}_{1}\mu_{1}{𝑇}_{1}+{𝑢}_{2}\mu_{2}{𝑇}_{2}\\ white{𝑁}^{{}^{'}}=\gamma 𝑁-\left(1-{𝑢}_{2}\right)\mu_{2}{𝑇}_{2}-\alpha 𝑁. \end{array}$$

We assume that the rate of return to the disease in each infertile women is the constant value of β, and the number of secondary sufferers is equal to the number of infertile women who were treated, but returned to this disease afterward during the course of treatment. At first, we assume that the number of people in the population is equal to the number of people susceptible to the disease. To find ${𝑅}_{0}$, we replace 𝑁(𝑡), and 𝑆(𝑡) with ${𝑆}_{0}$ and deduce the following system.

$$\left(\begin{array}{c} {𝐼}^{{}^{'}}\\ {𝑇}_{1}^{{}^{'}}\\ {𝑇}_{2}^{{}^{'}} \end{array}\right)=\left(\begin{array}{ccc} 0 & \beta \eta {𝑆}_{0} & \beta \zeta {𝑆}_{0}\\ 0 & 0 & 0\\ 0 & 0 & 0 \end{array}\right)\left(\begin{array}{c} 𝐼\\ {𝑇}_{1}\\ {𝑇}_{2} \end{array}\right)-\left(\begin{array}{ccc} \lambda +\alpha & 0 & 0\\ -𝑏\lambda & \mu_{1}+\alpha & 0\\ -(1-𝑏)\lambda & -\left(1-{𝑢}_{1}\right)\mu_{1} & \mu_{2}+\alpha \end{array}\right)\left(\begin{array}{c} 𝐼\\ {𝑇}_{1}\\ {𝑇}_{2} \end{array}\right).$$

This linear system is divided into two parts. The first matrix is shown by 𝐹 and is called returning matrix and the second matrix by 𝐾, which is called affected matrix. Thus, if

𝐹=0βη𝑆0βζ𝑆0000000 And K=α+λ00−𝑏λα+μ10−(1−𝑏)λ−(1−𝑢1)μ1α+μ2,

then 

$${𝐾}^{-1}=\left(\begin{array}{ccc} \frac{1}{\alpha +\lambda } & 0 & 0\\ \frac{𝑏\lambda }{\left(\alpha +\lambda \right)\left(\alpha +\mu_{1}\right)} & \frac{1}{\left(\alpha +\mu_{1}\right)} & 0\\ \frac{𝑏\lambda \left(1-{𝑢}_{1}\right)\mu_{1}+\left(\alpha +\mu_{1}\right)\lambda \left(1-𝑏\right)}{\left(\alpha +\lambda \right)\left(\alpha +\mu_{1}\right)\left(\alpha +\mu_{2}\right)} & \frac{\left(1-{𝑢}_{1}\right)\mu_{1}}{\left(\alpha +\mu_{2}\right)\left(\alpha +\mu_{1}\right)} & \frac{1}{\left(\alpha +\mu_{2}\right)} \end{array}\right).$$

Therefore, 

$${\mathrm{𝐹𝐾}}^{-1}=\left(\begin{array}{ccc} 𝐻 & 𝐺 & \frac{\beta \zeta {𝑆}_{0}}{\left(\alpha +\mu_{2}\right)}\\ 0 & 0 & 0\\ 0 & 0 & 0 \end{array}\right),$$

where 

$$𝐻=\frac{\beta \eta {𝑆}_{0}𝑏\lambda }{\left(\alpha +\lambda \right)\left(\alpha +\mu_{1}\right)}+\beta \zeta {𝑆}_{0}\left(\frac{𝑏\lambda \mu_{1}\left(1-{𝑢}_{1}\right)+\left(\alpha +\mu_{1}\right)\lambda \left(1-𝑏\right)}{\left(\alpha +\lambda \right)\left(\alpha +\mu_{1}\right)\left(\alpha +\mu_{2}\right)}\right)$$

and 

$$𝐺=\frac{\beta \eta {𝑆}_{0}}{\left(\alpha +\mu_{1}\right)}+\frac{\beta \zeta {𝑆}_{0}\left(1-{𝑢}_{1}\right)\mu_{1}}{\left(\alpha +\mu_{1}\right)\left(\alpha +\mu_{2}\right)}.$$

${𝑅}_{0}$ is the trace of ${\mathrm{𝐹𝐾}}^{-1}$ (5) Thus, 

$${𝑅}_{0}=\mathrm{𝑇𝑟𝑎𝑐𝑒}\left({\mathrm{𝐹𝐾}}^{-1}\right)=𝐻.$$

### Analysis of women's infertility based on asymptotical stability

Based on the biological hypothesis, we can deduce ${\mathrm{𝑆𝐼𝑇}}_{1}{𝑇}_{2}𝑅$ model.

Prior to the disease, the free equilibrium point is ${𝑄}_{0}=\left({𝑆}_{0},0,0,0,{𝑆}_{0}\right)$, and we have the following result (6).

**Theorem 2.1.1.** If ${𝑅}_{0}<1$ then:

a) If γ-α>0, then ${𝑄}_{0}$ is the free equilibrium point which is locally stable but it is not asymptotically stable.

b) If γ-α=0, then the free equilibrium point is unstable.

c) If γ-α<0, then ${𝑄}_{0}$ is locally asymptotically stable.

<statement> <title>Proof </title>

</statement>

(a) The linearized matrix of model ${\mathrm{𝑆𝐼𝑇}}_{1}{𝑇}_{2}𝑅$ at the point ${𝑄}_{0}$ is 

$$\left(\begin{array}{ccccc} -\alpha & 0 & -\beta \eta {𝑆}_{0} & -\beta \zeta {𝑆}_{0} & \gamma \\ 0 & -(\alpha +\lambda ) & \beta \eta {𝑆}_{0} & \beta \zeta {𝑆}_{0} & 0\\ 0 & 𝑏\lambda & -(\alpha +\mu_{1}) & 0 & 0\\ 0 & (1-𝑏)\lambda & \left(1-{𝑢}_{1}\right)\mu_{1} & -(\alpha +\mu_{2}) & 0\\ 0 & 0 & 0 & -\left(1-{𝑢}_{2}\right)\mu_{2} & \gamma -\alpha \end{array}\right).$$

The characteristic equation of this matrix is:

$$\left(-\alpha -\lambda^{{}^{'}}\right)\left(\gamma -\alpha -\lambda^{{}^{'}}\right)\{\left[\begin{array}{c} -\left(\left(\lambda +\alpha \right)+\lambda^{{}^{'}}\right)\left(\left(\mu_{1}+\alpha \right)+\lambda^{{}^{'}}\right)\left(\left(\mu_{2}+\alpha \right)+\lambda^{{}^{'}}\right)\\ +\beta {𝑆}_{0}\delta_{2}\lambda 𝑏\left(1-{𝑢}_{1}\right)\mu_{1} \end{array}\right]$$

$$-\left[-\beta {𝑆}_{0}\delta_{2}\lambda \left(1-𝑏\right)\lambda \left(\mu_{1}+\alpha \right)+\lambda^{{}^{'}}\right)-\beta {𝑆}_{0}\delta_{1}\lambda 𝑏\left(\left(\mu_{2}+\alpha \right)+\lambda^{{}^{'}}\right)]\}=0.$$

The roots of this equation are: 

$${\lambda^{{}^{'}}}_{1}=-\alpha \left(3.1\right)$$

$${\lambda^{{}^{'}}}_{2}=\gamma -\alpha \left(3.2\right)$$

If 𝑎0λ'3+𝑎1λ'2+𝑎2λ'+𝑎3=0.(3.3)

Then, for other roots equation (3.3) using Routh Hurwitz methods (7). As all of the parameters are positive, ${𝑎}_{1}=\left(3\alpha +\mu_{2}\right)+\lambda +\mu_{1}>0$. Moreover, ${𝑎}_{0}=1>0$.

We take ${𝑎}_{2}=\left[-𝑏\lambda \beta \eta {𝑆}_{0}+\left(\alpha +\lambda \right)\left(\alpha +\mu_{1}\right)+\left(\alpha +\mu_{2}\right)\left(\lambda +\alpha \right)+\left(\alpha +\mu_{1}\right)\left(\alpha +\mu_{2}\right)-\beta \zeta {𝑆}_{0}\left(1-𝑏\right)\lambda \right].$

Additionally, ${𝑎}_{3}=\left[-𝑏\lambda \beta \eta {𝑆}_{0}\left(\alpha +\mu_{2}\right)-𝑏\lambda \beta {𝑆}_{0}\zeta \mu_{1}\left(1-{𝑢}_{1}\right)+\left(\alpha +\mu_{1}\right)\left(\alpha +\mu_{2}\right)\left(\alpha +\lambda \right)-\left(\alpha +\mu_{1}\right)\beta \zeta {𝑆}_{0}\left(1-𝑏\right)\lambda \right].$

Since ${𝑅}_{0}<1$, then 

$$\left.\begin{array}{c} -\beta {𝑆}_{0}\eta \lambda 𝑏+\left(\lambda +\alpha \right)\left(\mu_{1}+\alpha \right)>0\\ -\beta {𝑆}_{0}\zeta \left(1-𝑏\right)\lambda +\left(\lambda +\alpha \right)\left(\mu_{2}+\alpha \right)>0 \end{array}\right\}\Rightarrow {𝑎}_{2}>0.\left(3.4\right)$$

Once again as ${𝑅}_{0}<1$, then $\left(\alpha +\lambda \right)\left(\alpha +\mu_{1}\right)\left(\alpha +\mu_{2}\right)>\beta \zeta {𝑆}_{0}\left(1-{𝑢}_{1}\right)\mu_{1}𝑏\lambda +\beta \eta {𝑆}_{0}𝑏\lambda \left(\alpha +\mu_{2}\right)+\beta \zeta {𝑆}_{0}\left(1-𝑏\right)\lambda^{2}\left(\alpha +\mu_{1}\right).$

Hence, 

$${𝑎}_{3}>0\left(3.5\right)$$

Moreover, $\Delta_{2}=\mathrm{𝑑𝑒𝑡}\left(\begin{array}{cc} {𝑎}_{1} & {𝑎}_{0}\\ {𝑎}_{3} & {𝑎}_{2} \end{array}\right)={𝑎}_{1}{𝑎}_{2}-{𝑎}_{0}{𝑎}_{3}$.

Since 

$$\begin{array}{c} \left(\alpha +\mu_{2}\right)\left(\left(\alpha +\lambda \right)\left(\alpha +\mu_{1}\right)-\beta \zeta {𝑆}_{0}\left(1-𝑏\right)\lambda \right)>0\\ \left(\lambda +\alpha \right)(\left(\alpha +\lambda \right)\left(\alpha +\mu_{1}\right)-\beta \eta {𝑆}_{0}𝑏\lambda )>0\\ \left(\alpha +\mu_{2}\right)\left(\left(\alpha +\lambda \right)\left(\alpha +\mu_{2}\right)-\beta \eta {𝑆}_{0}b\lambda \right)>0\\ \left(\alpha +\mu_{1}\right)\left(\left(\alpha +\lambda \right)\left(\alpha +\mu_{1}\right)-\beta \eta {𝑆}_{0}𝑏\lambda \right)>0\\ \left(\alpha +\lambda \right)(\left(\alpha +\lambda \right)\left(\alpha +\mu_{2}\right)-\beta \zeta {𝑆}_{0}\left(1-𝑏\right)\lambda )>0\\ \left(\alpha +\mu_{1}\right)\left(\left(\lambda +\alpha \right)\left(\mu_{2}+\alpha \right)-\beta {𝑆}_{0}\zeta \left(1-𝑏\right)\lambda \right)>0 \end{array}$$

Therefore, ${𝑎}_{1}{𝑎}_{2}-{𝑎}_{0}{𝑎}_{3}={\left(\alpha +\mu_{1}\right)}^{2}\left(\lambda +\alpha \right)+{\left(\alpha +\lambda \right)}^{2}\left(\mu_{2}+\alpha \right)+{\left(\alpha +\mu_{2}\right)}^{2}\left(\alpha +\lambda \right)+{\left(\alpha +\lambda \right)}^{2}\left(\mu_{1}+\alpha \right)+\left(\alpha +\mu_{2}\right)\left(\alpha +\lambda \right)\left(\alpha +\mu_{1}\right)+{\left(\alpha +\mu_{1}\right)}^{2}\left(\alpha +\mu_{2}\right)+b\lambda \beta \eta {𝑠}_{0}\left(\alpha +\alpha \right)-𝑏\lambda \beta \eta {𝑆}_{0}\left(\alpha +\mu_{1}\right)-\beta {𝑆}_{0}\zeta \lambda^{2}\left(1-𝑏\right)\left(\mu_{1}+\alpha \right)-\left(\alpha +\alpha \right)\beta \zeta {𝑆}_{0}\lambda \left(1-𝑏\right)+\beta {𝑆}_{0}\zeta \lambda 𝑏\left(1-{𝑢}_{1}\right)\mu_{1}+\beta {𝑆}_{0}\eta \lambda 𝑏\left(\mu_{2}+\alpha \right)>0\Rightarrow \Delta_{2}>0.$

$${𝑎}_{1}{𝑎}_{2}-{𝑎}_{0}{𝑎}_{3}>0.\left(3.6\right)$$

By using (3.5) and (3.6), we obtain 

$$\Delta_{3}=\mathrm{𝑑𝑒𝑡}\left(\begin{array}{ccc} {𝑎}_{1} & {𝑎}_{0} & 0\\ {𝑎}_{3} & {𝑎}_{2} & {𝑎}_{1}\\ 0 & 0 & {𝑎}_{3} \end{array}\right)={𝑎}_{3}\left({𝑎}_{1}{𝑎}_{2}-{𝑎}_{0}{𝑎}_{3}\right)>0.$$

Hence, by Hurwitz criterion, the real parts of the roots of (3.3) are all negative. If (3.2) is positive, then ${𝑄}_{0}$ is not locally asymptotically stable.

(b) If (3.2) is zero, then ${𝑄}_{0}$ is unstable.

(c) If ${\lambda^{{}^{'}}}_{2}=\gamma -\alpha <0$, then the free equilibrium point ${𝑄}_{0}$ is locally and asymptotically stable. (8, 9)

If γ>α, the number of patients who leave the clinic without result is less than the number of patients who enter the clinic. This demonstrates that considered treatment is useful. For a number of patients with Polycystic Ovary function, γ>α means local stability of free point; that is not asymptotically stable. If γ<α, it shows the asymptotical stability of system, and it states that patients who come to the center have an acute infertility problem. If γ=α, system is unstable, it means all the patients who come to the center leave there without any result. The evaulation of the treatment process depends on these three cases.

If 

$${𝑆}_{*}={\mathrm{𝐸𝑇}}_{1_{*}},$$

$${𝐼}_{*}=\frac{\left(\alpha +\mu_{1}\right)}{𝑏\lambda }{𝑇}_{1_{*}},$$

$${𝑇}_{2_{*}}=\frac{𝑏\left(1-{𝑢}_{1}\right)\mu_{1}+\left(1-𝑏\right)\left(\mu_{1}+\alpha \right)}{𝑏(\alpha +\mu_{2})}{𝑇}_{1_{*}},$$

$${𝑁}_{*}=\frac{\left(1-{𝑢}_{2}\right)\mu_{2}(𝑏\left(1-{𝑢}_{1}\right)\mu_{1}+\left(1-𝑏\right)\left(\mu_{1}+\alpha \right)}{\left(\lambda -\alpha \right)𝑏\left(\mu_{2}+\alpha \right)}{𝑇}_{1_{*}},$$

and 

$${𝑇}_{1_{*}}=\frac{\left(\alpha +\lambda \right)\left(\alpha +\mu_{1}\right)}{\lambda 𝑏(\beta \eta 𝐸+\beta \mathrm{𝐸𝐿}\zeta )}.$$

Where

$$𝐸=\frac{\gamma \lambda \left(1-{𝑢}_{2}\right)\mu_{2}\left(1-{𝑢}_{1}\right)\mu_{1}+\left(1-{𝑢}_{2}\right)\mu_{2}\left(1-𝑏\right)\left(\mu_{1}+\alpha \right)-\left(\lambda +\alpha \right)\left(\mu_{1}+\alpha \right)\left(\gamma -\alpha \right)\left(\mu_{2}+\alpha \right)}{\lambda b\left(\gamma -\alpha \right)\left(\mu_{2}+\alpha \right)},$$

$$𝐿=\frac{𝑏\left(1-{𝑢}_{1}\right)\mu_{1}+\left(1-𝑏\right)\left(\mu_{1}+\alpha \right)}{𝑏(\mu_{2}+\alpha )}.$$

Then, ${𝑄}_{*}=\left({𝑆}_{*},{𝐼}_{*},{𝑇}_{1_{*}},{𝑇}_{2_{*}},{𝑁}_{*}\right)$ shows the other equilibrium point. Since the ${𝑄}_{*}$ point is located in the interior of the positive space, we must have: 

λ>α,

$$\begin{array}{c} \gamma \lambda \left(1-{𝑢}_{2}\right)\mu_{2}\left(1-{𝑢}_{1}\right)\mu_{1}\\ +\left(1-{𝑢}_{2}\right)\mu_{2}\left(1-𝑏\right)\left(\mu_{1}+\alpha \right)\\ >\left(\lambda +\alpha \right)\left(\mu_{1}+\alpha \right)\left(\gamma -\alpha \right)\left(\mu_{2}+\alpha \right). \end{array}$$

### Sign stability of the dynamical model of women's infertility

When we model a biological phenomenon with differential equations, the values of the parameters used in them are error-prone. If we deal with a square matrix, then it is highly important to know how much the stability of this matrix depends on the elements of that matrix and how sensitive is it to the variations of the matrix elements. Therefore, in this section, using the following definitions and Theorems, we examine the sign stability of the system (1) at the equilibrium point of ${𝑄}_{*}$. To state the Theorem (2.2.3.), we use some concepts of graph theory as follows.

**Definition 2.2.1.**(10): An 𝑛×𝑛 square matrix A $=[{𝑎}_{\mathrm{𝑖𝑗}}]$ is said to be sign stable if every 𝑛×𝑛 square matrix B $=[{𝑏}_{\mathrm{𝑖𝑗}}]$ of the same sign pattern (i.e., ${\mathrm{𝑠𝑖𝑔𝑛𝑏}}_{\mathrm{𝑖𝑗}}={\mathrm{𝑠𝑖𝑔𝑛𝑎}}_{\mathrm{𝑖𝑗}}$ for all i; j = 1, 2,..., n) is a stable matrix.

For an 𝑛×𝑛 square matrix A $=[{𝑎}_{\mathrm{𝑖𝑗}}]$, we can obtain an undirected graph ${𝐺}_{𝐴}$, whose vertex set is V = 1,2,..., n and edges are {(𝑖,𝑗):𝑖≠𝑗;𝑎𝑖𝑗≠0≠𝑎𝑗𝑖;𝑖,𝑗=1,2,...,𝑛}. Also, a directed graph DA can also attach to A with the same vertex set and edges {(𝑖,𝑗):𝑖≠𝑗;𝑎𝑖𝑗≠0≠𝑎𝑗𝑖;𝑖,𝑗=1,2,...,𝑛}. A k-cycle of ${𝐷}_{𝐴}$ is a set of distinct edges of ${𝐷}_{𝐴}$ of the form: $\left\{\left({𝑖}_{1},{𝑖}_{2}\right),\left({𝑖}_{2},{𝑖}_{3}\right),....,\left({𝑖}_{𝑘-1},{𝑖}_{𝑘}\right),\left({𝑖}_{𝑘},{𝑖}_{1}\right)\right\}$: Let ${𝑅}_{𝐴}=\left\{𝑖:{𝑎}_{\mathrm{𝑖𝑖}}\neq 0\right\}\subset 𝑉$, which are the numbers for them the corresponding element in the main diagonal of the matrix is not zero. An ${𝑅}_{𝐴}$-coloring of ${𝐺}_{𝐴}$ is a partition of its vertices into two sets, black and white (one of which may be empty). Such that each vertex in ${𝑅}_{𝐴}$ is black, no black vertex has precisely one white neighbor, and each white vertex has at least one white neighbor. A V $-{𝑅}_{𝐴}$ complete matching is a set M of pairwise disjoint edges of ${𝐺}_{𝐴}$ such that the set of vertices of the edges in M contains every vertex in V $-{𝑅}_{𝐴}$. By applying this concepts, we are now able to state the following Theorem.

**Theorem 2.2.2.**(10): An 𝑛×𝑛 real matrix A $=[{𝑎}_{\mathrm{𝑖𝑗}}]$ is sign stable if it satisfies the following conditions:

(i) ${𝑎}_{\mathrm{𝑖𝑗}}\leq 0$ for all i, j;

(ii) ${𝑎}_{\mathrm{𝑖𝑗}}{𝑎}_{\mathrm{𝑗𝑖}}\leq 0$ for all 𝑖≠𝑗;

(iii) The directed graph ${𝐷}_{𝐴}$ has no k-cycle for k ≥3;

(iv) In every ${𝑅}_{𝐴}$-coloring of the undirected graph ${𝐺}_{𝐴}$ all vertices are black; and

(v) The undirected graph ${𝐺}_{𝐴}$ admits a ${𝑅}_{𝐴}$ complete matching.

**Theorem 2.2.3.** If γ>α, then the matrix model ${\mathrm{𝑆𝐼𝑇}}_{1}{𝑇}_{2}𝑅$ at the equilibrium ${𝑄}_{*}$ is not sign stable.

<statement> <title>Proof </title>

</statement>

The matrix of the linearize system model of ${\mathrm{𝑆𝐼𝑇}}_{1}{𝑇}_{2}𝑅$ at the equilibrium ${𝑄}_{*}$ is given by

$$𝐴=\left(\begin{array}{ccccc} -\alpha -\beta (\eta {𝑇}_{1_{*}}+\zeta {𝑇}_{2_{*}}) & 0 & -\beta \eta {𝑆}_{*} & -\beta \zeta {𝑆}_{*} & \gamma \\ \beta (\eta {𝑇}_{1_{*}}+\zeta {𝑇}_{2_{*}}) & -(\alpha +\lambda ) & \beta \eta {𝑆}_{*} & \beta \zeta {𝑆}_{*} & 0\\ 0 & 𝑏\lambda & -(\alpha +\mu_{1}) & 0 & 0\\ 0 & \left(1-𝑏\right)\lambda {𝑃}_{*} & \left(1-{𝑢}_{1}\right)\mu_{1} & -(\alpha +\mu_{2}) & 0\\ 0 & 0 & 0 & -\left(1-{𝑢}_{2}\right)\mu_{2} & \gamma -\alpha \end{array}\right).$$

Where ${𝑃}_{*}={𝐼}_{*}.$ Theorem (2.2.2.) requires 

$$𝐵=\left(\begin{array}{ccccc} -1 & 0 & -1 & -1 & 1\\ 0 & -1 & 1 & 1 & 0\\ 0 & 1 & -1 & 0 & 0\\ 0 & 1 & 1 & -1 & 0\\ 0 & 0 & 0 & -1 & 1 \end{array}\right).$$

Which is not sign stable. Since ${𝑎}_{55}>0$, then Theorem 2.2.2. implies that the matrix 𝐴 is not sign stable. ⊔⊓

Theorem 2.2.3. implies that in ${\mathrm{𝑆𝐼𝑇}}_{1}{𝑇}_{2}R$ model at equilibrium point ${𝑄}_{*}$ is not sign stable. This is a positive case from the perspective of the disease treatment, since the sign stability of ${\mathrm{𝑆𝐼𝑇}}_{1}{𝑇}_{2}𝑅$ model is equivalent to the return of disease, which has not been reversed for a long time; as a result, the problem of infertility and reproduction still remains for a high percentage of the population.

### Reversibility of the infertility cycle with the PCOS factor in an ${\mathrm{𝑆𝐼𝑇}}_{1}{𝑇}_{2}𝑅$ model is not intermittent

The infertility treatment with 𝑃𝐶𝑂𝑆 initiates as a course of treatment with Clomiphene Citrate and Gonadotropin in some menstrual cycles, which, by the failure of medical treatment, changes the treatment with the 𝐼𝑉𝐹 therapy cycle. These patients will stay in that therapy course until they achieve the desirable result from it. From a medical point of view, this therapeutic cycle will not include the return to medical treatment during its treatment course. In this section, we prove that in system (1), the return of infertility is not circular in the cycle of the disease with the 𝑃𝐶𝑂𝑆 factor. In other words, the disease has no circulation.

First, we state the Stoke's Theorem.

**Theorem 2.3.1. (Stoke's Theorem)** (9): Suppose that 𝑀 is a bordered, oriented, compact, 𝑘 dimensional Manifold, and also suppose that ω is a smooth 𝑘-1 form on 𝑀. In this case: 

$$\int_{\partial 𝑀}\omega =\int_{𝑀}𝑑\omega$$

Where ∂𝑀, as described earlier, is an oriented border.

Now, with the help of Stoke's Theorem, we state the Theorem on the closed circuits in this model.

**Theorem 2.3.2.** The${\mathrm{𝑆𝐼𝑇}}_{1}{𝑇}_{2}𝑅$ model does not have a closed circuit.

<statement> <title>Proof </title>

</statement>

Assumption by contradiction: If there is a closed circuit like C with the rotation period of T, then we consider the Manifold M as a region in ${𝑅}^{6}$ with the border C, and assume ω as a 5-form on M, which is defined as follows:

$\omega =\left(\gamma 𝑁-\alpha 𝑆-\beta 𝑆\left(\eta {𝑇}_{1}+\zeta {𝑇}_{2}\right)\right)\mathrm{𝑑𝐼}⋀{\mathrm{𝑑𝑇}}_{1}⋀{\mathrm{𝑑𝑇}}_{2}⋀\mathrm{𝑑𝑁}⋀\mathrm{𝑑𝑅}+(-\lambda 𝐼-\alpha 𝐼+\beta 𝑆\left(\eta {𝑇}_{1}+\zeta {𝑇}_{2}\right)\mathrm{𝑑𝑆}⋀{\mathrm{𝑑𝑇}}_{1}⋀{\mathrm{𝑑𝑇}}_{2}⋀\mathrm{𝑑𝑁}⋀\mathrm{𝑑𝑅}+\left(-\mu_{1}{𝑇}_{1}-\alpha {𝑇}_{1}+\lambda \mathrm{𝐼𝑏}\right)\mathrm{𝑑𝑆}⋀\mathrm{𝑑𝐼}⋀{\mathrm{𝑑𝑇}}_{2}⋀\mathrm{𝑑𝑁}⋀\mathrm{𝑑𝑅}+\left(-\mu_{2}{𝑇}_{2}-\alpha {𝑇}_{2}+\left(1-{𝑢}_{1}\right)\mu_{1}{𝑇}_{1}+\left(1-𝑏\right)\lambda 𝐼\right)\mathrm{𝑑𝑆}⋀\mathrm{𝑑𝐼}⋀{\mathrm{𝑑𝑇}}_{1}⋀\mathrm{𝑑𝑁}⋀\mathrm{𝑑𝑅}+\left(\gamma 𝑁-\left(1-{𝑢}_{2}\right)\mu_{2}{𝑇}_{2}-\alpha 𝑁\right)\mathrm{𝑑𝑆}⋀\mathrm{𝑑𝐼}⋀{\mathrm{𝑑𝑇}}_{1}⋀{\mathrm{𝑑𝑇}}_{2}⋀\mathrm{𝑑𝑅}+\left(-\alpha 𝑅+{𝑢}_{1}\mu_{1}{𝑇}_{1}+{𝑢}_{2}\mu_{2}{𝑇}_{2}\right)\mathrm{𝑑𝑆}⋀\mathrm{𝑑𝐼}⋀{\mathrm{𝑑𝑇}}_{1}⋀{\mathrm{𝑑𝑇}}_{2}⋀\mathrm{𝑑𝑁}.$

Therefore,

$𝑑\omega =\left(-6\alpha -\beta \left(\eta {𝑇}_{1}+\zeta {𝑇}_{2}\right)-\mu_{1}-\mu_{2}\right)\mathrm{𝑑𝑆}⋀\mathrm{𝑑𝐼}⋀{\mathrm{𝑑𝑇}}_{1}⋀{\mathrm{𝑑𝑇}}_{2}⋀\mathrm{𝑑𝑁}⋀\mathrm{𝑑𝑅}.$

Hence, ∫𝑀𝑑ω<0 and ∫∂𝑀=𝐶ω=0. However, it is concluded from Stoke's Theorem that $0=\int_{𝑀}𝑑\omega <\int_{\partial 𝑀=𝐶}\omega =0$and this is a contradiction. Therefore, the system (1) has no closed circuit; in other words, the reversibility of the therapeutic cycle of this disease is not intermittent.

### Numerical solutions of the model $SIT_{1}T_{2}R$

In this section, we solve ${\mathrm{𝑆𝐼𝑇}}_{1}{𝑇}_{2}𝑅$ model numerically via Rung-Kutta method (11). First, we define the following functions:

$${𝑓}_{1}\left(𝑡,𝑆,𝐼,{𝑇}_{1}\;{𝑇}_{2},𝑁,𝑅\right)=\gamma 𝑁-\alpha 𝑆-\beta 𝑆\left(\eta {𝑇}_{1}+\zeta {𝑇}_{2}\right)$$

$${𝑓}_{2}\left(𝑡,𝑆,𝐼,{𝑇}_{1}\;{𝑇}_{2},𝑁,𝑅\right)=-\lambda 𝐼-\alpha 𝐼+\beta 𝑆\left(\eta {𝑇}_{1}+\zeta {𝑇}_{2}\right)$$

$${𝑓}_{3}\left(𝑡,𝑆,𝐼,{𝑇}_{1}\;{𝑇}_{2},𝑁,𝑅\right)=-\mu_{1}{𝑇}_{1}-\alpha {𝑇}_{1}+\lambda \mathrm{𝐼𝑏}$$

$${𝑓}_{4}\left(𝑡,𝑆,𝐼,{𝑇}_{1}\;{𝑇}_{2},𝑁,𝑅\right)=-\mu_{2}{𝑇}_{2}-\alpha {𝑇}_{2}+\left(1-{𝑢}_{1}\right)\mu_{1}{𝑇}_{1}+\left(1-𝑏\right)\lambda 𝐼$$

$${𝑓}_{5}\left(𝑡,𝑆,𝐼,{𝑇}_{1}\;{𝑇}_{2},𝑁,𝑅\right)=\gamma 𝑁-\left(1-{𝑢}_{2}\right)\mu_{2}{𝑇}_{2}-\alpha 𝑁$$

$${𝑓}_{6}\left(𝑡,𝑆,𝐼,{𝑇}_{1}\;{𝑇}_{2},𝑁,𝑅\right)=-\alpha 𝑅+{𝑢}_{1}\mu_{1}{𝑇}_{1}+{𝑢}_{2}\mu_{2}{𝑇}_{2}$$

Hence,${𝑆}_{0},{𝐼}_{0},{{(𝑇}_{1})}_{0}\;{{(𝑇}_{2})}_{0},{𝑁}_{0},$and${𝑅}_{0}$ are the initial conditions.

We take$ℎ=\frac{𝑏-𝑎}{𝑀}$, where 𝑀>0 and${𝑡}_{𝑗}=𝑎+\mathrm{𝑗𝑘}$ for 𝑗=0,1,2,⋯,𝑀.

For 𝑖=1,2,3,4,5,6, we take 

$${𝐾}_{1,𝑖}={ℎ}_{𝑖}\left({𝑡}_{𝑗},{𝑆}_{𝑗},{𝐼}_{𝑗},{{𝑇}_{1}}_{𝑗},{{𝑇}_{2}}_{𝑗},{𝑁}_{𝑗},{𝑅}_{𝑗}\right),$$

$${𝐾}_{2,𝑖}={ℎ}_{𝑖}\left(t_{j}+\frac{h}{2},S_{j}+\frac{K_{1,1}}{2},I_{j}+\frac{K_{1,2}}{2},{T_{1}}_{j}+\frac{K_{1,3}}{2},{T_{2}}_{j}+\frac{K_{1,4}}{2},N_{j}+\frac{K_{1,5}}{2},R_{j}+\frac{K_{1,6}}{2}\right),$$

$$K_{3,i}=hf_{i}\left(t_{j}+\frac{h}{2},S_{j}+\frac{K_{2,1}}{2},I_{j}+\frac{K_{2,2}}{2},{T_{1}}_{j}+\frac{K_{2,3}}{2},{T_{2}}_{j}+\frac{K_{2,4}}{2},N_{j}+\frac{K_{2,5}}{2},R_{j}+\frac{K_{2,6}}{2}\right),\mathrm{and}$$

$$K_{4,i}=hf_{i}\left(t_{j}+h,S_{j}+K_{3,1},I_{j}+K_{3,2},{T_{1}}_{j}+K_{3,3},{T_{2}}_{j}+K_{3,4},N_{j}+K_{3,5},R_{j}+K_{3,6}\right).$$

We put${𝑊}_{1,𝑗}={𝑆}_{𝑗},{𝑊}_{2,𝑗}={𝐼}_{𝑗}$,${𝑊}_{3,𝑗}={{𝑇}_{1}}_{𝑗},{𝑊}_{4,𝑗}={{𝑇}_{2}}_{𝑗},{𝑊}_{5,𝑗}={𝑁}_{𝑗},$ and${𝑊}_{6,𝑗}={𝑅}_{𝑗}$. So, 

$$W{i.j+1}_{}=W_{i,j}+\frac{1}{6}\left(K_{1,i}+2K_{2,i}+2K_{3,i}+K_{4,i}\right)$$

If 𝑀=50 and 𝑆0=15,𝐼0=30,(𝑇1)0=27 (𝑇2)0=12,𝑁0=104 and 𝑅0=20, then we have the following graphs for the solutions.

**Figure 7:**
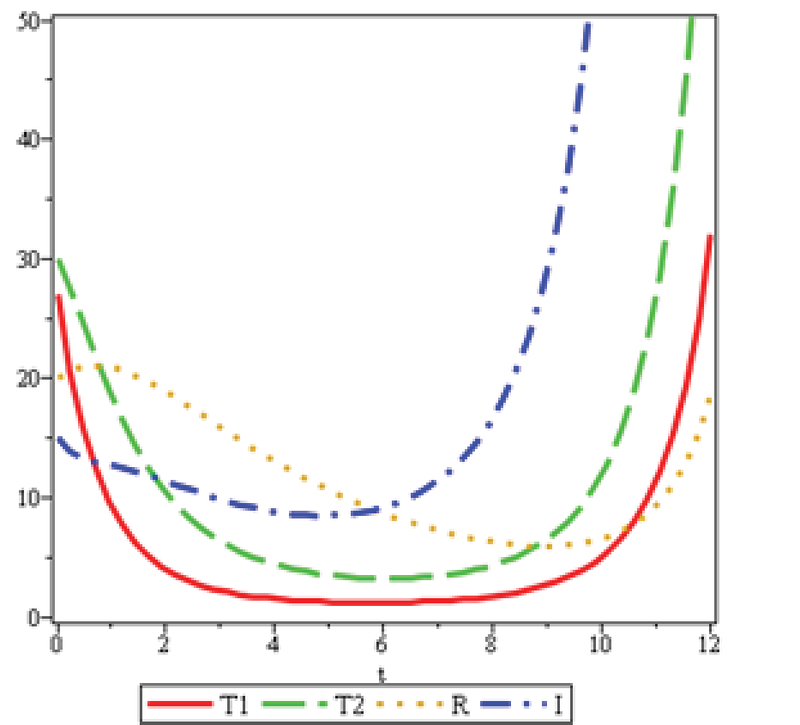
$\lambda =0.47,b=0.34,\eta =0.47,\mu_{1}=0.9,u_{1}=0.04,\zeta =0.16$, $\mu_{2}=0.92,u_{2}=0.29,\alpha =0.3$ and γ=0.5.

**Figure 8:**
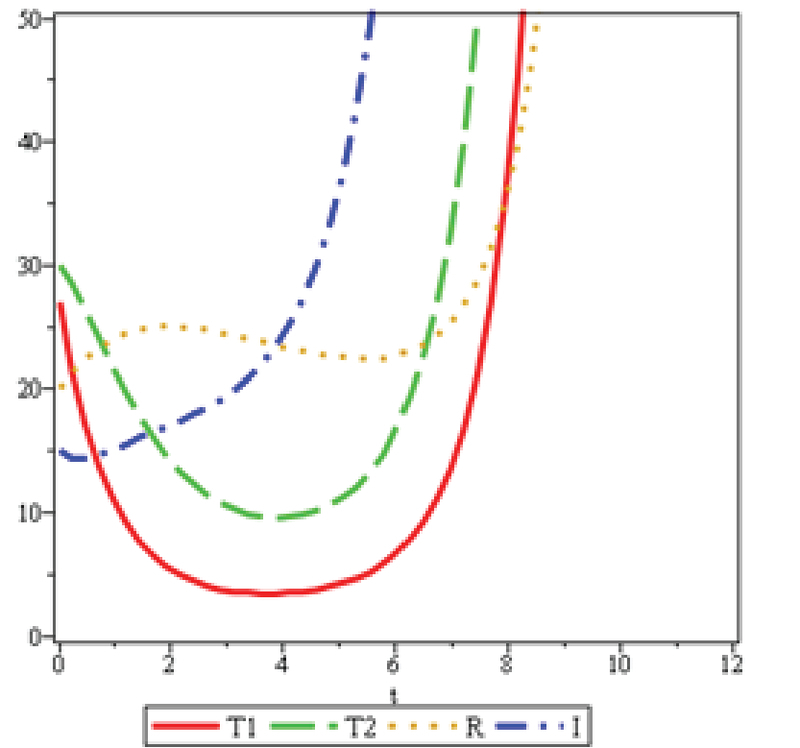
$\lambda =0.47,b=0.34,\eta =0.47,\mu_{1}=0.9,u_{1}=0.04,\zeta =0.16,\mu_{2}=0.92,u_{2}=0.29,\alpha =0.16,$ and γ=0.23.

Figures 7 and 8 are, respectively, considered for infertile women aged 20-25 and 25-30 with female factor and ovarian reserve of more than 3.5, based on the statistical data of Yazd Infertility Center. In Figure 8, α is twice as in Figure 7; therefore, the number of the patients treated by ${𝑇}_{2}$ in Figure 8 in its lowest amount (minimum) is half the number of patients treated by ${𝑇}_{2}$ in Figure 7. The treatment duration of the patients in Figure 8 is approximately 1.5 times longer than that in patients in Figure 7. Moreover, ${𝑇}_{1}$ and ${𝑇}_{2}$ in Figure 7, and at the end of the cycle, have an incremental mode. The treatment speed of ${𝑇}_{2}$ is more than that of ${𝑇}_{1}$, since the increase of age, the ovarian reserve decreases, and the medical treatment with clomiphene citrate and gonadotropin will no more be responsive. Therefore, the treatment with IVF ${𝑇}_{2}$ is recommended to these patients. The evidences can exactly be observed in Figure 8. Similarly, the recovery speed of R in the Figure 7 reaches its minimum with no longer be responsive, to the extent that the minimum number of the improved patients in Figure 8 is approximately 0.25 of the number of the improved patients in Figure 7. In Figures 7 and 8, when R decreases, the number of patients increases.

**Figure 9:**
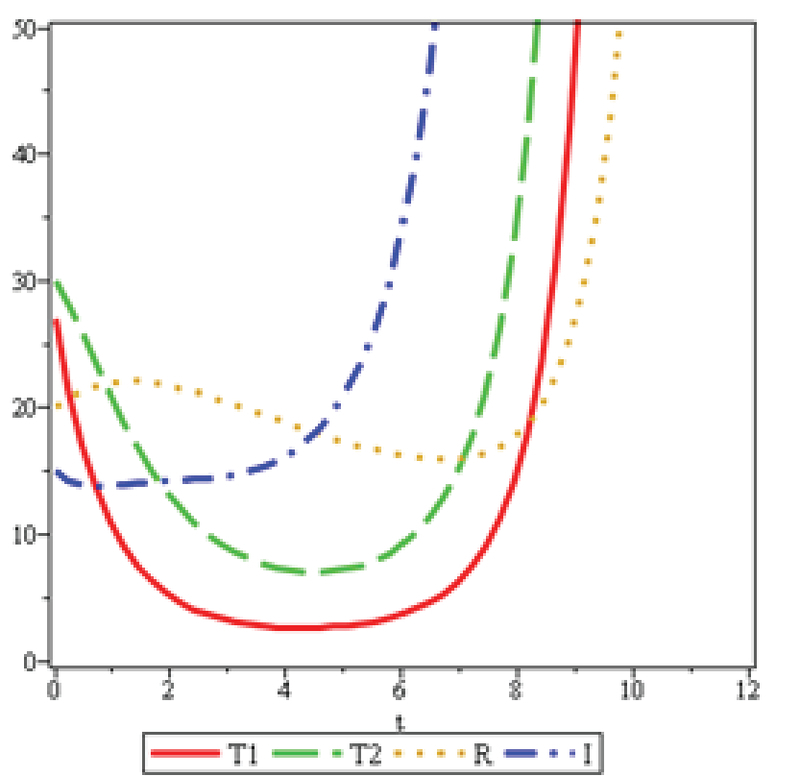
$\lambda =0.47,b=0.34,\eta =0.47,\mu_{1}=0.9,u_{1}=0.04,\zeta =0.08$, $\mu_{2}=0.96,u_{2}=0.19,\alpha =0.16,$ and γ=0.23.

Figure 9 has been reviewed for the patients with the ovarian reserve less than 3.5. In Figure 7, ${𝑢}_{2}$ is twice more than the ${𝑢}_{2}$ in Figure 9. Therefore, the number of the improved patients R in Figure 8 at its lowest value is almost twice as much as than the number of improved individuals in Figure 9. The length of treatment period in Figure 8 is approximately 1.5 times more than the length of treatment in Figure 9, since the speed of referral to the clinic, shown byγ, with respect to the ovarian reserve in Figure 8, is four times more than that in Figure 8.

## 3. Discussion

The analysis of mathematical models depends on the sustainability of the disease in the community. In this model, the population volume is considered constant at a constant time and provides a good estimate for short periods with similar patients' conditions. Information obtained from the Yazd Infertility Center confirms that the ovarian reserve decreases as the age increases, thus, the number of patients attending IVF treatment increases, as seen clearly in Figures 7 and 8.

## 4. Conclusions

In this research, ${\mathrm{𝑆𝐼𝑇}}_{1}{𝑇}_{2}𝑅$ model is considered to cure infertility in couples. It is proved that the disease free equilibrium point ${𝑄}_{0}$ for${\mathrm{𝑆𝐼𝑇}}_{1}{𝑇}_{2}𝑅$ model is locally stable and it is not asymptotically stable, when${𝑅}_{0}<1$. Here, ${𝑅}_{0}$ is the number of patients who during treatment come across with the illness for the second time, and return to the clinic for secondary treatment. Furthermore, by using the Rung-Kutta method, we solved the model for achieving numerical solution. Considering the numerical results, the patients with ovariarn reserve less than 3.5 with two category of age range 20-25 and 25-30, it is shown that as age increases the ovarian reserve decreases, hence, Clomiphene Citrate and Gonadotropin treatments are not responsive. Obviously, IVF treatment is recommended in this group of patients. The main achievement of this study was that more ovarian reserve resulted in more cured infertile patients.

##  Conflict of Interest 

The authors declare that they have no competing interests.
